# Early Adaptive Humoral Immune Responses and Virus Clearance in Humans Recently Infected with Pandemic 2009 H1N1 Influenza Virus

**DOI:** 10.1371/journal.pone.0022603

**Published:** 2011-08-23

**Authors:** Chao Qiu, Di Tian, Yanmin Wan, Wanju Zhang, Chenli Qiu, Zhaoqin Zhu, Ruiqi Ye, Zhigang Song, Mingzhe Zhou, Songhua Yuan, Bisheng Shi, Min Wu, Yi Liu, Shimin Gu, Jun Wei, Zhitong Zhou, Xiaoyan Zhang, Zhiyong Zhang, Yunwen Hu, Zhenghong Yuan, Jianqing Xu

**Affiliations:** 1 Shanghai Public Health Clinical Center, Institutes of Biomedical Sciences, Fudan University, Shanghai, China; 2 Key Laboratory of Medical Molecular Virology of Ministry of Education/Health, Shanghai Medical College, Fudan University, Shanghai, China; 3 State Key Laboratory for Infectious Disease Prevention and Control, China CDC, Beijing, China; 4 Yuncheng CDC, Yuncheng, China; Statens Serum Institute, Denmark

## Abstract

Few studies on the humoral immune responses in human during natural influenza infection have been reported. Here, we used serum samples from pandemic 2009 H1N1 influenza infected patients to characterize the humoral immune responses to influenza during natural infection in humans. We observed for the first time that the pandemic 2009 H1N1 influenza induced influenza A-specific IgM within days after symptoms onset, whereas the unit of IgG did not changed. The magnitude of influenza A-specific IgM antibodies might have a value in predicting the rate of virus clearance to some degree. However, the newly developed IgM was not associated with hemagglutination inhibition (HI) activities in the same samples but correlated with HI activities of subsequently collected sera which were mediated by IgG antibodies, indicating that IgM was critical for influenza infection and influences subsequent IgG antibody responses. These findings provide new important insights on the human immunity to natural influenza infection.

## Introduction

Adaptive humoral immunity is an essential component of immune responses to different microbe infections including influenza. Different isotypes of immunoglobulin (Ig) constitute the adaptive humoral immunity to influenza and play different roles in protection and pathogenesis [1]. Influenza specific IgG is superior to other isotypes in neutralization activity and is able to effectively contain progeny virus when used as a regiment of immunotherapy in immunodeficient mice [2]. Secreted IgM in acute phase of infection also plays important role in protection from influenza virus. Both natural IgM produced by B-1 cells in the absence of exposure to virus and antigen induced IgM secreted by B-2 cells after antigen stimulation nonredundantly contribute to immune protection from infection of influenza virus [3]. However, most of these data have been collected from mice infection model. Infection of Influenza usually lasts for a week and most infected people with mild symptoms recover within weeks without hospitalization, which renders it difficult to collect samples during the early acute infection from clinical settings. So far no observation intensively monitoring early humoral responses to the pandemic or seasonal influenza infection in humans has been reported.

On April 15 and 17, 2009, two epidemiologically unlinked cases of new swine influenza A in children residing in California were reported [4]. Sequence analysis revealed that genetic characterization of the virus was a combination of gene segments derived from triple reassortant North American and Eurasian swine lineages [5], [6], [7], [8], [9]. Within weeks this novel strain of influenza spread globally by human-to-human transmission [10]. The outbreak of influenza infection had received much attention from public than ever before in last decades and many strategies had been employed to prevent its spread. On May 24, 2009, we identified a Chinese working in Australia as the first case of 2009 pandemic H1N1 infection in Shanghai, which marked the beginning of sporadic 2009 H1N1 cases in east China. To prevent community epidemic, all patients indentified from May 24^th^ to July 9^th^ in Shanghai were quarantined and treated in Shanghai Public Health Clinical Center until the viral loads of their nasopharyngeal swab became undetectable. This opportunity allowed us to collect sera of influenza infected patients longitudinally and initiated experiments aiming at characterizing early humoral immune responses to influenza.

## Materials and Methods

### Patients and Serum samples

131 patients hospitalized in Shanghai Public health Clinical Center (SHAPHC) received diagnostic of 2009 H1N1 infection, which had been confirmed by mean of real time PCR. Nasopharyngeal swab was obtained daily for monitoring the change of the concentration of viral genome, in terms of viral load. Among these H1N1 patients, 73 subjects had serum samples on two or more days. The majority of these patients (99.6%) received oseltamivir according to H1N1 treatment protocol and had been stayed in hospital for 3–9 days. A set of outpatient sera was gathered for non-influenza serological testing in September of 2008 as non-exposed control. Sera of Human respiratory syncytial virus, parainfluenza and adenovirus infected subjects were collected from laboratories participating regional respiratory disease surveillance program. Sera of HIV cohort were obtained in September of 2009 and in June of 2010.

### Ethics Statement

Written informed consents were obtained from all 2009 Influenza A (H1N1) infected subjects right after they were admitted into hospital and from all participants in HIV cohorts. Storage sera collected from outpatients in 2008 during non-flu season and sera of human respiratory syncytial virus, parainfluenza and adenovirus infected subjects were fully delinked from patient personal information by the removal of all labels from samples before they were tested per the requirement from Ethics Committee of SHAPHC. The overall study was reviewed and proved by the Ethics Committee of SHAPHC.

### Measurement of anti-influenza A antibodies by ELISA

Commercial ELISA kits were used to assess the units of IgG (Minneapolis, Minnesota, IBL, Cat# IB79251) and IgM (Minneapolis, Minnesota, IBL, Cat# IB79252) antibodies to influenza A following manufacture's instruction. Briefly, 100 µL each of the 1∶100 diluted samples and the standards were added respectively into the wells and incubated at room temperature for 60 minutes. After three washes with washing solution, the wells were incubated with peroxide-conjugated anti-human IgG or anti-human IgM antibodies at room temperature for 30 minutes. Plates were then washed for another three times and 100 µL TMB substrate was pipetted and incubated at room temperature for approximately 20 minutes. After development of a blue dye in the wells, reaction was terminated by the addition of 100 µL stop solution. In IgM ELISA, to eliminate interference from hyper-immune levels of IgG or rheumatoid factors, sera were pretreated with RF adsorbent (Minneapolis, Minnesota, IBL, Cat# 79000) according to manufacture's instruction. Samples with unit of IgM ≥12 were considered positive as suggested by manufacture's instruction.

### Hemagglutination-inhibition (HAI) assays

Serum sample were treated with receptor destroying enzyme (RDE) (St. Louis, MO, Sigma, Cat# C8772) to inactivate non-specific inhibitors. Then serially diluted 2-fold into U-bottom 96-well microtiter plates starting at a dilution of 1∶10 and stopping at 1∶320 in volume of 50 µL. 50 µL of virus adjusted to approximately 8 HA units was added. The plates were covered and incubated at room temperature for 30 min followed by the addition of 1% gninea pig erythrocytes (RBCs) in PBS. The plates were mixed by agitation, covered, and allowed to set for 60 min at room temperature. The HI titer was determined by the reciprocal of the last dilution that contained non-agglutinated RBCs. 2009 H1N1 vaccinated serum was used as positive control and serum from naïve mouse raised under specific pathogen free circumventions was used as negative serum controls. Samples with HI titers ≥1∶40 were considered seropositive. The virus strain used was A/Shanghai/37T/2009(H1N1) (SH37T) (Genebank ID: ACS27776∼ACS27785), which shares 99.47% similarity of HA protein sequence (M8L, S220T, V428C) to A/California/04/2009.

### Removal of IgM and IgG

Five serum samples with both high unit of IgM and high titer of HI were pooled. IgG was removed using Protein G - sepharose beads. Briefly, 200 µL of Protein G –sepharose beads was washed for three times with ice cooled PBS. At the final wash, the supernatant was aspired after centrifuging and 100 µL of pooled serum was added. Incubated the serum-beads mixture at 4°C under rotary agitation for 1 hour, centrifuged the tube and keep the supernatant as IgG depleted serum. All steps were repeated for three times to remove abundant IgG. In the case of IgM depletion, instead of Protein G - sepharose beads, anti-human IgM antibody was incubated with Protein G - sepharose beads prior to addition of pooled serum.

### Statistics analysis

Geometric mean titers (GMTs) were calculated by assigning a titer of 5 to samples no HI antibody was detected at the starting dilution (titer<10). Mann-Whitney U test was used to calculate *p*-value when compared GMT between two groups. Wilcoxon signed rank test was used to calculate *p*-value when compared GMT between paired data. Spearman's rank correlation coefficient was used to calculate the correlation between the unit of IgM and the titer of HI. Log-rank (Mantel-Cox) test was used to compare survival curves and to calculated hazard ratio and its 95% confidence intervals (CI) of the association of factors. Chi-square test was used to calculate p value, odds ratios (OR) and 95% confidence intervals (CI) of the association of factors in contingency tables. In the box plot, the whiskers represent 10–90^th^ percentile.

## Results

### 1. Early IgM responses in individuals recently infected with pandemic H1N1 influenza viruses

**Figure 1:**
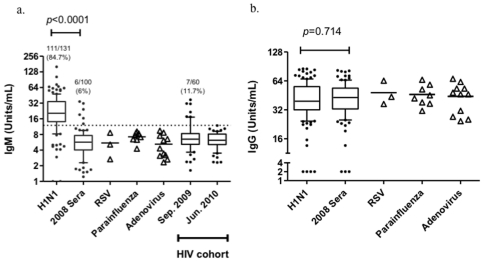
Influenza A specific IgM and IgG in serum samples from 2009 pandemic H1N1 infected patients and non-influenza related sources. The units of IgM (a) and IgG (b) antibodies to influenza A in sera were determined by commercial ELISA kits. 131 pandemic 2009 H1N1 influenza infected samples, 100 samples collected in Sep. 2008, 3 acute RSV infected samples, 8 acute parainfluenza infected samples, 11 acute adenovirus infected samples and samples from 60 HIV+ subjects taken in Sep. 2009 and Jun. 2010 were also used in this experiment. In the box plot, the whiskers represent 10–90th percentile.

As the first immunoglobulin class produced during microbial infection, IgM is widely accepted as the first line of primary immune response and diminishes quickly in few days of infection. Firstly we examine whether the influenza A-specific IgM in sera could reflect the history of recent exposure to influenza A. Influenza A-specific IgM and IgG were quantified in samples of 131 pandemic 2009 H1N1 infected patients on the 1^st^ or 2^nd^ day after they were admitted into hospital. As showed in figure 1a, among 131 samples of 2009 H1N1 infected patients who were finally confirmed by real time PCR, 111 (84.7%) samples were positive for anti-influenza A IgM, whereas only 6 (6%) out of 100 serum samples colleted in September of 2008 that was not a flu pandemic season were positive (OR = 86.95, 95%CI [34.10–220.39], *p*<0.0001). These data gave rise to the sensitivity at 94.9% with 95%CI from 89.2% to 98.1% and the specificity at 82.5% with 95%CI from 74.2% to 88.9%. To further determine the specificity of the IgM assay, a panel of serum samples from other acute respiratory viral infections including RSV, parainfluenza and adenovirus were also tested and all of them were negative for influenza A-specific IgM. Besides those samples, we used residual serum samples taken from a HIV cohort study to validate whether Influenza A-specific IgM is a surrogate marker for recent exposure to H1N1 influenza. Of 60 HIV+ samples, 7 (11.7%) colleted in the epidemic wave of 2009 H1N1 influenza (Sep. 2009) were positive for influenza A-specific IgM, whereas none was positive in samples collected in June of 2010. In contrast to IgM, high influenza A -specific IgG level was detected in almost all individuals (Fig. 1b), indicating that almost all individuals were infected by influenza A or were vaccinated with seasonal flu vaccine before.

**Figure 2:**
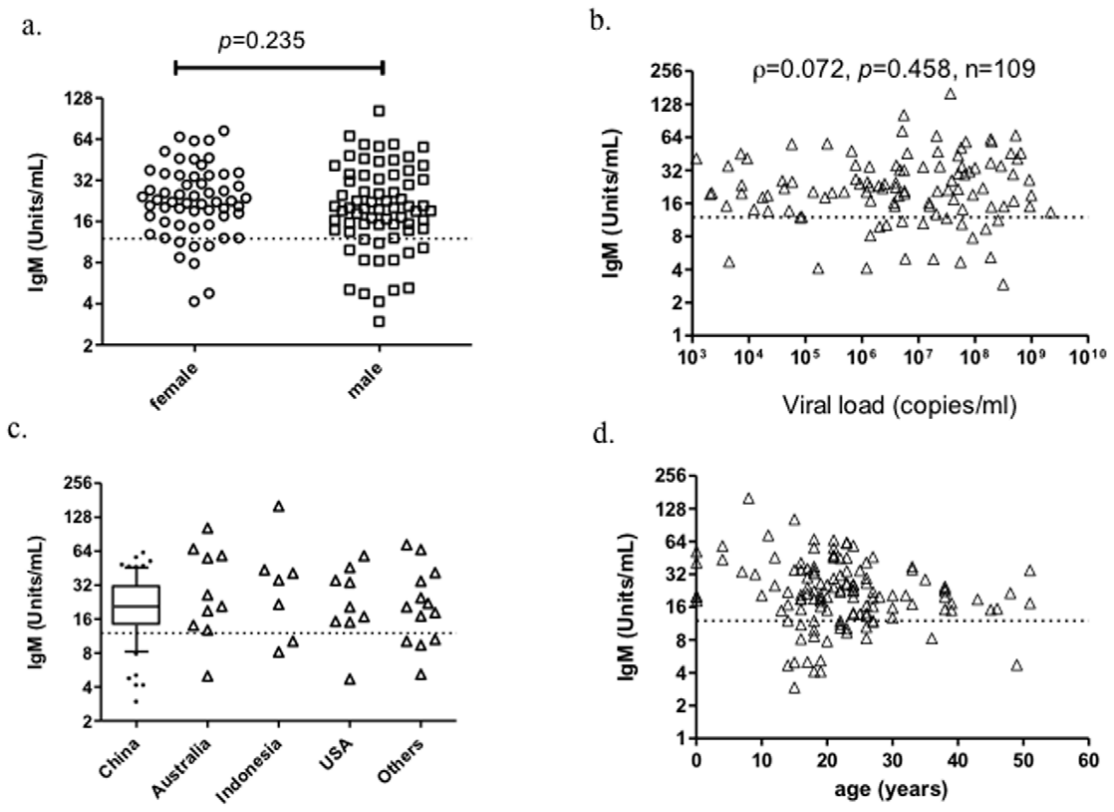
Cross sectional analysis of the associations between IgM and genders, viral loads, nationalities and ages. a) Mann-Whitney U test was used to test the difference of IgM between 58 female and 71 male H1N1 infected patients; b) The association between 109 paired data of IgM and viral loads in nasopharyngeal swab was analyzed using spearman rank correlation; c) The difference of grouped samples by nationality was tested by Kruskal-Wallis test; d) Age distribution of IgM. All data were derived from samples of the 2nd day post symptom onset.

**Figure 3:**
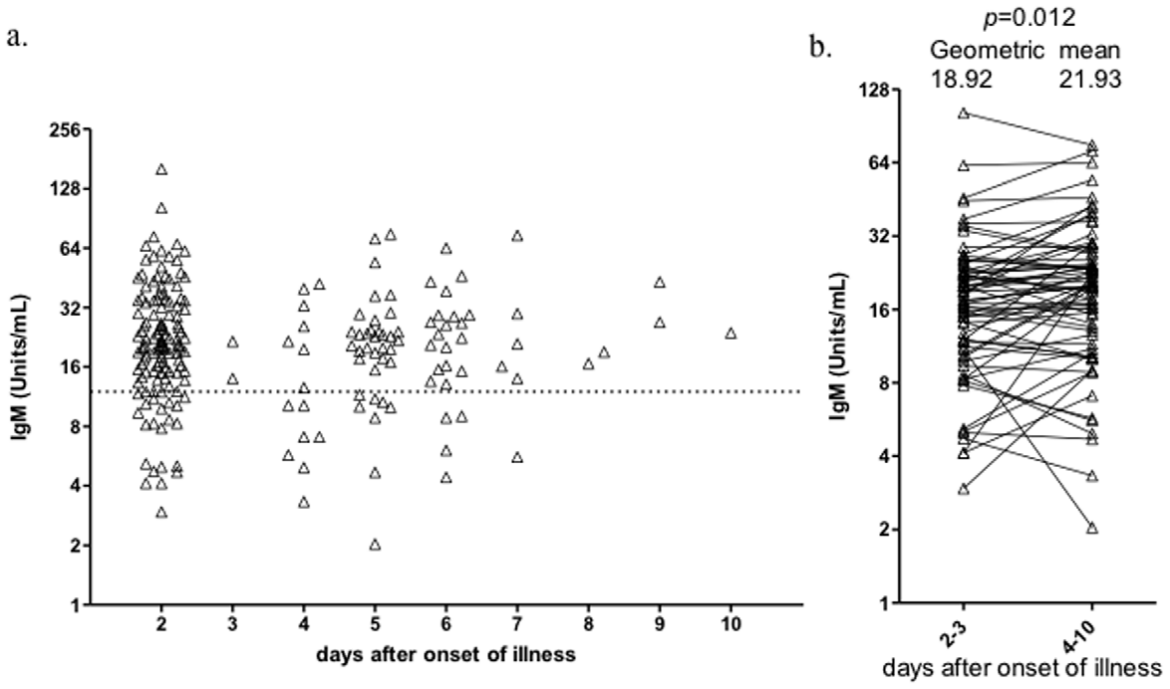
Time course of IgM in week of H1N1 infection. a) IgM grouped by days since of illness; b) Longitudinal kinetic of IgM in 73 paired subjects who had seral samples with multiple time-points.

We next examined whether the development of IgM antibodies was associated with patient gender, viral loads, genetic background and ages (Fig. 2). The unit of IgM was not significantly associated with sex (Mann-Whitney U test, *p* = 0.235) (Fig. 2a). Spearman's rank correlation coefficient between the unit of IgM and viral load of nasopharyngeal swab was 0.072, suggesting no correlation (*p* = 0.458) (Fig. 2b). There was no significant difference of the unit of IgM when samples were grouped by nationality (Kruskal-Wallis test, *p* = 0.720) (Fig. 2c). Slight higher unit of IgM was observed in age group of 5–10 years than others, but it did not reach statistic significance due to few child cases (28.36 VS 18.73, *p* = 0.107) (Fig. 2d). The longitudinal kinetic of IgM in population was also examined. The percentage of positive IgM was slightly increased from 84.9% on the 2^nd^ and 3^th^ day of symptom onset to 92.7% on the 4–10^th^ day of symptom onset and the geometric mean increased from 18.92 to 21.93 (*p* = 0.012) (Fig. 3).

Overall, our data suggested that anti-influenza A IgM could be newly developed within days after exposure to 2009 H1N1 influenza. Patient gender, viral loads, genetic background and ages have no significantly role in the development of anti-influenza A IgM antibodies.

### 2. IgM plays a minor role in virus clearance

**Figure 4:**
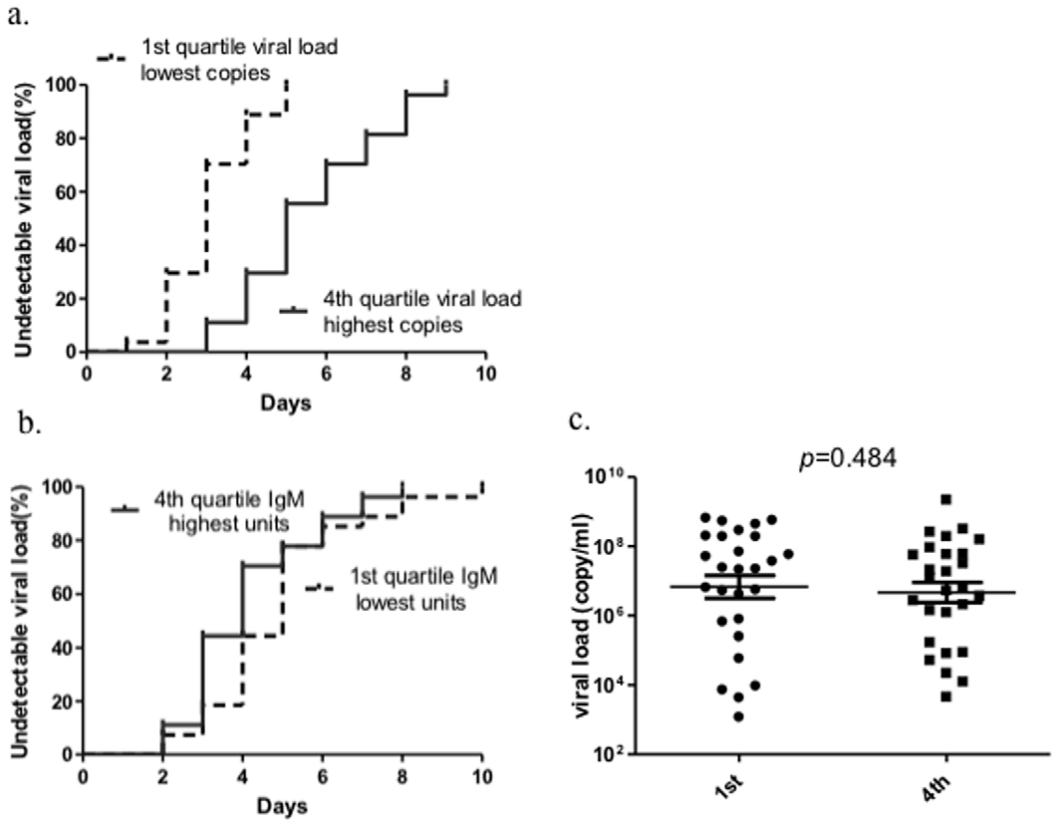
Initial viral load in nasopharyngeal swab and serum IgM affect virus clearance. Proportions of patients with undetectable viral loads in days since admitted to hospital were graphed with the days post symptom onset. Patients were divided into quartiles by viral load (a) or unit of IgM (b), 1st and 4th quartiles were showed. Each quartile had 27 subjects; c) The viral loads of patients in groups of 1st and 4th quartiles of IgM unit were compared.

Since secreted IgM is an important component in protective responses to influenza in mouse model, we performed an analysis to understand whether IgM level was correlated with virus clearance at this early course of human natural infection. The outcome event was defined as viral load of nasopharyngeal swab became undetectable. The patients were divided into quartiles according to either the unit of IgM or viral loads on the 2^nd^ day of symptom onset. As showed in Fig. 4a, the group with highest copies of viral load had been stayed in hospital for a median of 5 days and the ones with lowest viral loads for 3 days (hazard ratio = 8.665, 95%CI [3.948–19.02], *p*<0.0001), the 2^nd^ and 3^rd^ quartiles of initial viral loads intercalated between the 1^st^ and 4^th^ quartiles (Figure S1a), suggesting the initial viral loads in nasopharynx is prognostic marker for virus clearance. We then tested whether the IgM is a prognostic marker for virus clearance. Interestingly, the group with highest units of IgM cleared the virus fairly quicker than the ones with lowest units (hazard ratio = 0.582, 95%CI [0.296–0.835], *p* = 0.041) (Fig. 4b), despite viral loads were comparable between these two groups (Fig. 4c). This was much manifested between day 3 and 5 after admission. However, given the comparison was performed between the highest and lowest IgM groups and the difference was relatively minor, we concluded that IgM may only play a minor role in viral clearance during human influenza infection, this was also evidenced in the groups with modest units of IgM (the 2^nd^ and 3^rd^ quartiles of IgM units) in which only minor differences were observed in their clearance days from those two extreme groups (Figure S1b).

### 3. Hemagglutination-inhibition activities in individuals recently infected with pandemic H1N1 influenza viruses

**Figure 5:**
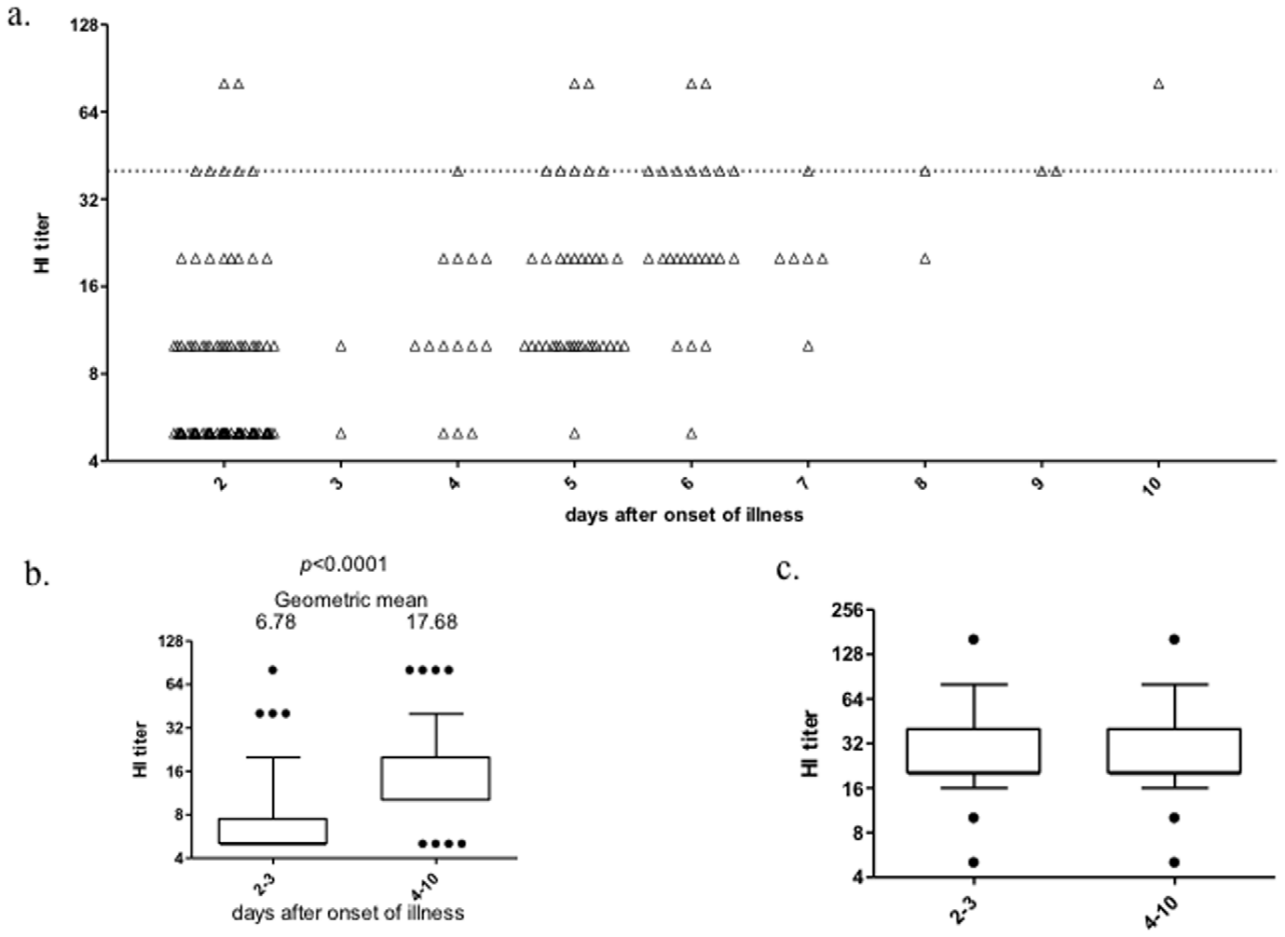
Time course of HI titers against homologous and heterologous virus strains in week of H1N1 infection. a) HI titers against 2009 H1N1 grouped by days since of illness; b) Longitudinal kinetic of HI titers against 2009 H1N1 in 73 subjects. Paired two samples from each subject, including one was collected within 3 days after the illness onset and the other was collected during day 4–10, were determined for their HI titers in parallel and compared; c) HI titers against H3 in 25 paired subjects infected with 2009 H1N1.

To understand whether IgM was correlated with hemagglutination-inhibition (HI) activities at this early course of infection, we performed HI assay. The virus strain used was SH37T, a primary virus isolated from a 30 years old male patient in our own laboratory. As shown in Fig. 5a (left), 7 out 131 sera on the 2^nd^ day of symptom onset reached HI titers ≥40 and 2 of them even reached titers at 80 whereas the majority had undetectable HI activities which was lower than 10. In week of symptom onset, the HI activity continuously elevated and most increased to titer at 10 or 20, a fraction of them reached titer >40, which was particularly on 5 days after admission (Fig. 5a). Since some of patients were released before the collection of their second samples during day 4–10, in order to exclude the bias resulted from unbalanced sample sizes, we further compared HI titers in all paired samples from 73 subjects, including one was collected within 3 days after illness onset and the other was collected during day 4–10 from each subject, and demonstrated that HI titers in samples collected during day 4–10 were significantly higher than those in samples collected within 3 days (Fig. 5b). The HI activities against seasonal H3 were not changed, suggesting no cross-reaction relationship between 2009 pandemic H1N1 and seasonal H3 for their HI activities (Fig. 5c).

**Figure 6:**
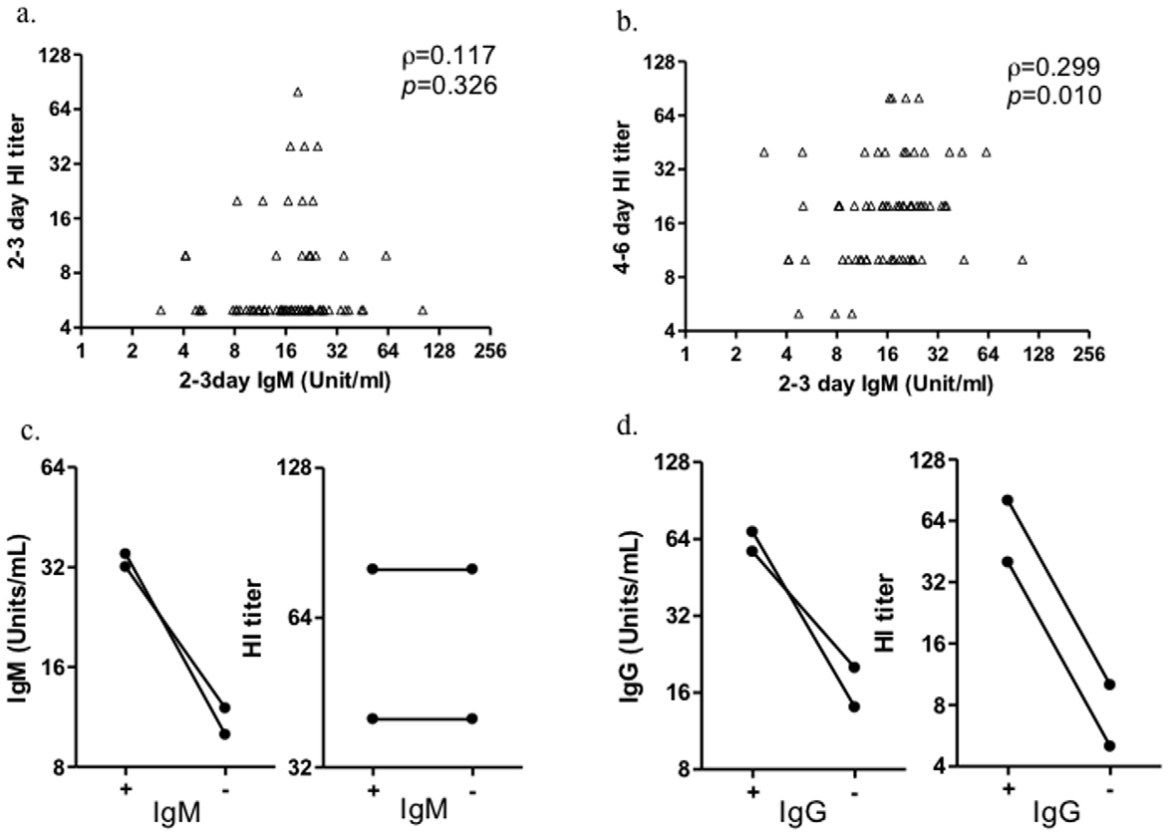
Association between IgM and HI titers and the contribution of IgM to HI activities. The association of early IgM and HI titers in early days (a) or late days (b) since influenza infection was shown. The depletion of IgM (c) or IgG (d) affect HI titers against 2009 H1N1 infeluenza virus.

Interestingly, the unit of IgM in serum collected at admission was not significantly associated with HI titer in the same samples (Spearman's rank correlation rho = 0.117, *p* = 0.326, Fig. 6a) but correlated with HI titer of serum samples colleted before discharge (Spearman's rank correlation rho = 0.299, *p* = 0.010, Fig. 6b). Interestingly, HI titers of sera from patients who cleared the virus was higher than that in non-cleared subjects though statistical significance was not reached (Figure S2).To test the how much the different subtypes of immunoglobulins contribute to HI activities, IgM and IgG in pooled sera were removed respectively. When IgM was depleted from the sera, the HI activities still remained (Fig. 6c). In contrast, the removal of IgG resulted in a significant decline of HI activities (Fig. 6d).

These data suggest that early developed influenza A-specific IgM antibodies are unable to exert HI activities but associated with the subsequent development of influenza A-specific IgG antibodies which could exert HI activities and might be associated with viral clearance; Good influenza A-specific IgM responses at very early phase could be considered as a marker of better influenza A-specific immune response in host and thereby associated with the early virus clearance.

## Discussion

The pandemic of 2009 H1N1 influenza infection has posed a challenge to global public health and emphasized the importance to conduct influenza surveillance in general population. A biomarker only transiently appeared during early influenza infection will be extremely useful for identifying newly influenza-infected individuals, which will entitle the opportunity to further characterize the newly infection and thereby monitor the general population. Since IgM antibody is the first antibodies elicited in immune responses following infection, we investigated the kinetics of IgM antibodies during this 2009 H1N1 influenza prevalence. Our data showed that among 2009 H1N1 influenza infected patients ∼85% individuals developed IgM antibodies within days and this number was further increased to >90% if a longitudinal observation was performed; In contrast, only 6% individuals showed IgM positive among samples collected from population at a non-flu season. These data gave rise to a sensitivity at 95% and a specificity at 82.5%. Since it is known that a sporadic influenza A virus infection may occasionally occur during non-flu season, the 6% Influenza A IgM positive cases in 2008 non-flu season sera samples may represent the real newly Influenza A virus infection, we expected that the specificity for this IgM assay could be above 82.5%. This speculation was further confirmed by the observation that none positive cases were identified among samples from RSV-, parainfluenza-, adenovirus- and HIV-infected cases at non-influenza season. However, the sensitivity and the specificity of IgM assay will subject to be further adjusted in a large scale study. Although previous report in mice model has indicated that immunoglobulin M antibodies could be induced during influenza virus infection (3), this report is for the first time to observe that influenza A specific IgM could appear within days after natural influenza infection in humans.

The high sensitivity and the good specificity of IgM assay entitled IgM as a good biomarker for the surveillance of new influenza infection. IgM ELISA has several advantages over real time PCR [11], [12]. Firstly, ELISA is a test routinely used in clinical laboratory that doesn't require specific instruments, therefore, a regularly clinical laboratory is capable to undertake this detection task. Secondly, unlike viral load in nasopharyngeal swab which is quickly declined when medication is taken, IgM response usually persists for a few days regardless medical intervention. Thirdly, by the virtue of IgM response it is more important to monitor virus carriers without obviously symptoms and signs. In addition, the influenza A specific IgM based assay could remain as an effective tool for surveillance during circulations of new influenza A recombinants whereas PCR may miss the detection of the new recombinants since their primers are non-specific for the new recombinants. Under this circumstance, IgM assay could be employed as the first line tool to monitor the surveillance and PCR plus sequencing could be used thereafter as a tool for genotyping.

Rapid antigen test for Influenza A has been used as a complementary diagnostic tool in clinical settings, however, this assay has been generally considered as an assay with low sensitivity. Indeed, a recent clinical study showed that only 11.1% samples are tested as positive by rapid antigen test in 144 PCR-confirmed positive samples and the paramount feature for rapid test-positive was high virus concentration (13). In contrast, 85–92% out of 131 PCR-positive samples was positive in IgM assay in our study, suggesting that IgM assay may represent a much more sensitive assay than rapid antigen test. In addition, IgM response lasts longer than viral antigen, therefore, IgM test could remain as positive in samples which the viral replication starts to wane and thereby serve as a better complementary diagnostic tool in clinical settings.

Since previous report demonstrated that IgM responses could play an important protective role in influenza viral infection in mice model (3), therefore, we determined whether the higher IgM level could be associated with quicker viral clearance. Interestingly, our data showed that the group with highest units of IgM cleared virus fairly quicker than the one with lowest units with the maximized difference between day 3 and 5 after admission. However, a significant difference was only observed in the highest and lowest IgM groups and the difference was relatively minor, we concluded that IgM may only play a minor role in natural influenza viral clearance.

Since the efficacy of this early immune response to clear viruses is highly relevant to clinical prognosis, we quantified the HI activities of sera against a primary 2009 H1N1 influenza strain. Unexpectedly, though we observed that the vast majority of 2009 H1N1 influenza infected individuals developed IgM antibodies within days after infection and a good influenza A-specific IgM responses is a prognostic marker for viral clearance, we observed that nearly all 2009 H1N1 influenza infected individuals have low or undetectable HI activities in the early days of infection and good HI activities usually appeared a week after infection; Our additional experiments demonstrated that HI activities was conferred by the influenza A-specific IgG antibodies which was developed later than IgM, these data could explain the delayed appeared HI activities. Interestingly, we further identified that early good IgM responses were associated with subsequent good HI activities which is mediated by influenza A-specific IgG antibodies, in another word, early good IgM responses is associated with subsequent good IgG antibody responses, indicating that there may exist a innate linkage between early influenza A-specific IgM responses and subsequent IgG antibody responses, and both may be developed from pre-existing cross-reactive influenza A-specific immune memory pools in host and thereby correlated with the early virus clearance. In addition, it is known that T-cell responses play a role at the early stage of infection, a complete understanding on the immune responses and on their association with the viral clearance at the early phase will need to quantify the Influenza A-specific T cell responses in combination with humoral responses.

## Supporting Information

Figure S1 Viral clearance analysis of patients divided into groups by quartiles of either viral loads (a) or IgM (b).
(TIFF)

Figure S2 HI titer in sera of patients cleared the virus is higher than those of non-cleared.
(TIFF)

## References

[1] Strugnell RA, Wijburg OL (2010). The role of secretory antibodies in infection immunity.. Nat Rev Microbiol.

[2] Palladino G, Mozdzanowska K, Washko G, Gerhard W (1995). Virus-neutralizing antibodies of immunoglobulin G (IgG) but not of IgM or IgA isotypes can cure influenza virus pneumonia in SCID mice.. J Virol.

[3] Baumgarth N, Herman OC, Jager GC, Brown LE, Herzenberg LA (2000). B-1 and B-2 cell-derived immunoglobulin M antibodies are nonredundant components of the protective response to influenza virus infection.. J Exp Med.

[4] (2009). Swine influenza A (H1N1) infection in two children–Southern California, March-April 2009.. MMWR Morb Mortal Wkly Rep.

[5] Garten RJ, Davis CT, Russell CA, Shu B, Lindstrom S (2009). Antigenic and genetic characteristics of swine-origin 2009 A(H1N1) influenza viruses circulating in humans.. Science.

[6] Smith GJ, Vijaykrishna D, Bahl J, Lycett SJ, Worobey M (2009). Origins and evolutionary genomics of the 2009 swine-origin H1N1 influenza A epidemic.. Nature.

[7] Dawood FS, Jain S, Finelli L, Shaw MW, Lindstrom S (2009). Emergence of a novel swine-origin influenza A (H1N1) virus in humans.. N Engl J Med.

[8] Shinde V, Bridges CB, Uyeki TM, Shu B, Balish A (2009). Triple-reassortant swine influenza A (H1) in humans in the United States, 2005–2009.. N Engl J Med.

[9] Zhang H, Chen L (2009). Possible origin of current influenza A H1N1 viruses.. Lancet Infect Dis.

[10] (2009). World Health Organization. New influenza A (H1N1) virus: global epidemiological situation.. Wkly Epidemiol Rec.

[11] Faix DJ, Sherman SS, Waterman SH (2009). Rapid-test sensitivity for novel swine-origin influenza A (H1N1) virus in humans.. N Engl J Med.

[12] McCracken J (2009). Diagnosis of swine-lineage influenza A (H1N1) virus infection.. Lancet.

