# Supplementary figures and images for: Early Adaptive Humoral Immune Responses and Virus Clearance in Humans Recently Infected with Pandemic 2009 H1N1 Influenza Virus
Source: PLoS One. 2011 Aug 23;6(8):e22603. doi: 10.1371/journal.pone.0022603 (PMC3160288; doi:10.1371/journal.pone.0022603)

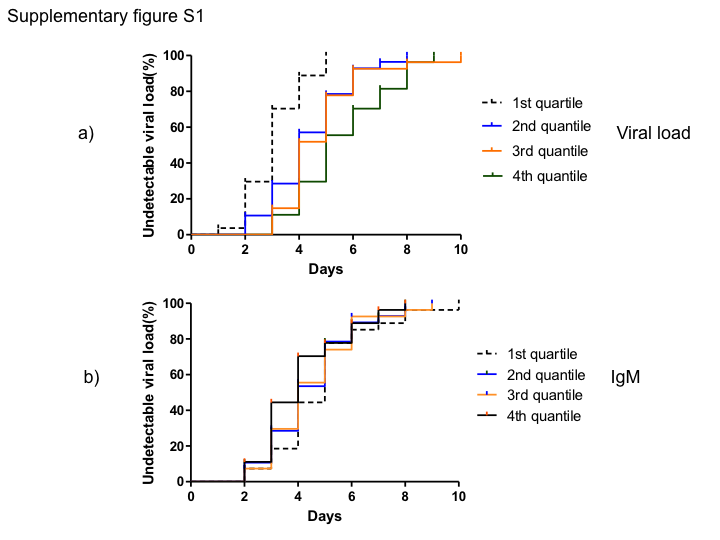

Supplement: Figure S1 — Viral clearance analysis of patients divided into groups by quartiles of either viral loads (a) or IgM (b). (TIFF) [file pone.0022603.s001.tiff]

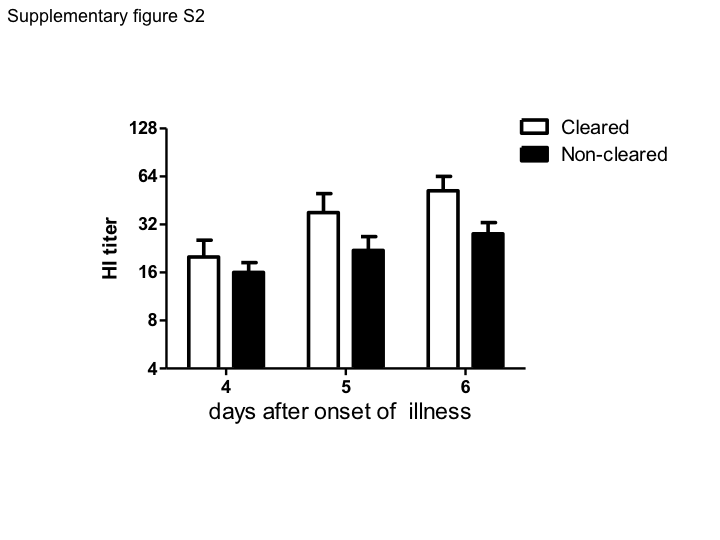

Supplement: Figure S2 — HI titer in sera of patients cleared the virus is higher than those of non-cleared. (TIFF) [file pone.0022603.s002.tiff]
